# Splenic Arterial Pulsatility Index to Predict Hepatic Fibrosis in Hemodialysis Patients with Chronic Hepatitis C Virus Infection

**DOI:** 10.3390/jcm12052020

**Published:** 2023-03-03

**Authors:** Chen-Hua Liu, Yu-Jen Fang, Chun-Jen Liu, Tung-Hung Su, Shang-Chin Huang, Tai-Chung Tseng, Jo-Hsuan Wu, Pei-Jer Chen, Jia-Horng Kao

**Affiliations:** 1 Department of Internal Medicine, National Taiwan University Hospital, Taipei 100225, Taiwan; 2 Hepatitis Research Center, National Taiwan University Hospital, Taipei 100225, Taiwan; 3 Department of Internal Medicine, National Taiwan University Hospital, Yun-Lin Branch, Douliou 640203, Taiwan; 4 Graduate Institute of Clinical Medicine, National Taiwan University College of Medicine, Taipei 100233, Taiwan; 5 Department of Internal Medicine, National Taiwan University Hospital Bei-Hu Branch, Taipei 108206, Taiwan; 6 Department of Medical Research, National Taiwan University Hospital, Taipei 100225, Taiwan; 7 Hamilton Glaucoma Center, Shiley Eye Institute and Viterbi Family Department of Ophthalmology, University of California, San Diego, CA 92039, USA

**Keywords:** hepatitis C virus, hepatic fibrosis, duplex Doppler ultrasonography, splenic arterial pulsatility index, noninvasive diagnosis, liver stiffness, transient elastography

## Abstract

The clinical utility of the splenic arterial pulsatility index (SAPI), a duplex Doppler ultrasonographic index, to predict the stage of hepatic fibrosis in hemodialysis patients with chronic hepatitis C virus (HCV) infection remains elusive. We conducted a retrospective, cross-sectional study to include 296 hemodialysis patients with HCV who underwent SAPI assessment and liver stiffness measurements (LSMs). The levels of SAPI were significantly associated with LSMs (Pearson correlation coefficient: 0.413, *p* < 0.001) and different stages of hepatic fibrosis as determined using LSMs (Spearman’s rank correlation coefficient: 0.529, *p* < 0.001). The areas under receiver operating characteristics (AUROCs) of SAPI to predict the severity of hepatic fibrosis were 0.730 (95% CI: 0.671–0.789) for ≥F1, 0.782 (95% CI: 0.730–0.834) for ≥F2, 0.838 (95% CI: 0.781–0.894) for ≥F3, and 0.851 (95% CI: 0.771–0.931) for F4. Furthermore, the AUROCs of SAPI were comparable to those of the fibrosis index based on four parameters (FIB-4) and superior to those of the aspartate transaminase (AST)-to-platelet ratio index (APRI). The positive predictive value (PPV) for ≥F1 was 79.5% when the Youden index was set at 1.04, and the negative predictive values (NPVs) for ≥F2, ≥F3, and F4 were 79.8%, 92,6%, and 96.9%, respectively, when the maximal Youden indices were set at 1.06, 1.19, and 1.30. The diagnostic accuracies of SAPI with the maximal Youden index for a fibrosis stage of ≥F1, ≥F2, ≥F3, and F4 were 69.6%, 67.2%, 75.0%, and 85.1%, respectively. In conclusion, SAPI can serve as a good noninvasive index in predicting the severity of hepatic fibrosis in hemodialysis patients with chronic HCV infection.

## 1. Introduction

Despite the adoption of universal precautions and blood safety, chronic hepatitis C virus (HCV) infection remains a significant health problem in hemodialysis patients. While the global prevalence of HCV infection is about 0.7%, the prevalence of HCV infection ranges from 4% to 20% in hemodialysis patients [1,2,3,4,5,6]. Hemodialysis patients with chronic HCV infection have higher hepatic and extrahepatic morbidity and mortality than those without chronic HCV infection [7,8,9]. In contrast, the long-term prognosis is improved once HCV is eradicated with effective antiviral treatment [10].

In the era of interferon (IFN), treatment uptake of HCV is low because the treatment response and tolerance are far from satisfactory [11,12,13]. The advent of IFN-free direct-acting antivirals (DAAs) after 2014 has made a paradigm shift in the care of hemodialysis patients with chronic HCV infection because the efficacy and safety are excellent with DAA treatment. Numerous clinical trials and real-world studies have indicated that more than 95% of hemodialysis patients with chronic HCV infection can achieve a sustained virologic response (SVR) with a short course of DAAs [14,15,16,17,18,19,20]. Although the stage of hepatic fibrosis does not significantly affect the overall response rates in hemodialysis patients with HCV receiving DAAs, an accurate diagnosis of the stage of hepatic fibrosis is still mandatory to assist in optimizing clinical decisions [21].

Percutaneous liver biopsy is the gold standard to assess the severity of hepatic fibrosis in patients with HCV infection. However, it is an invasive procedure that may cause deaths, major bleeding, biliary injuries, or pain [22]. The risk of bleeding in hemodialysis patients following percutaneous liver biopsy ranges from 1.3% to 5.9%, much higher than the risk of 0.16% in nonuremic patients [23,24,25]. Moreover, the biopsy specimens are prone to sampling and interpretation variations [26]. Therefore, using simple and easily accessible noninvasive indices to determine the therapeutic and surveillance plans is paramount for hemodialysis patients with chronic HCV infection [27].

Duplex Doppler ultrasonography (DDU) is an easily accessible noninvasive tool to evaluate the vascular dynamics in various organs. Clinically, physicians can perform DDU at a routine gray-scale ultrasonography screening. Prior studies have shown that the splenic arterial pulsatility index (SAPI), which measures the arterial resistance by placing the Doppler cursor within the main branches of the splenic artery at the splenic hilum, is highly correlated with the severity of hepatic fibrosis and portal hypertension in patients with chronic HCV infection, taking percutaneous liver biopsy and hepatic vein catheterization as the reference standards [28,29,30,31]. However, data regarding the value of SAPI to predict the stage of hepatic fibrosis in hemodialysis patients with chronic HCV infection are limited. We aimed to conduct a cross-sectional study to evaluate the clinical utility of SAPI to stage hepatic fibrosis in this special population, taking transient elastography (TE), which generates a shear wave in the liver tissue to directly determine the liver stiffness, to be the reference standard [32].

## 2. Materials and Methods

### 2.1. Patients

We conducted a retrospective, cross-sectional study to include hemodialysis patients with chronic HCV infection at the National Taiwan University Hospital (NTUH) and NTUH Yun-Lin Branch who underwent a liver stiffness measurement (LSM) with TE (FibroScan^®^, Echosens, Paris, France) and SAPI with duplex Doppler ultrasonography (Aplio 500^®^, Canon Medical Systems Incorporation, Tokyo, Japan) between January 2010 and June 2022. Hemodialysis patients were defined as those who had an estimated glomerular filtration (eGFR) rate <15 mL/min/1.73 m^2^ using the chronic kidney disease–epidemiology collaboration (CKD–EPI) equation and were on maintenance dialysis through vascular routes [33,34,35]. Chronic HCV infection was defined as patients who presented detectable HCV antibodies (anti-HCV; Abbott HCV EIA 2.0, Abbott Laboratories, Abbott Park, IL, USA) and quantifiable serum HCV RNA (Cobas TaqMan HCV Test v2.0, Roche Diagnostics GmbH, Mannheim, Germany, lower limit of quantification [LLOQ]: 15 IU/mL) for 6 months or more. Patients were excluded from the study if they had hepatitis B virus (HBV) or human immunodeficiency virus (HIV) coinfection, decompensated cirrhosis (Child-Pugh B or C), a history of hepatocellular carcinoma (HCC), a failed or unreliable LSM with TE, or a failed SAPI assessment due to splenectomy.

### 2.2. Study Design

We collected baseline demographic data, including age, sex, history of HCC, and body mass index (BMI). Blood tests, including hemogram, serum albumin, total bilirubin, aspartate transaminase (AST), alanine transaminase (ALT), creatinine, anti-HCV, anti-HIV (Abbott Architect HIV Ag/Ab Combo, Abbott Laboratories, Abbott Park, IL, USA), HBV surface antigen (Abbott Architect HBsAg qualitative assay, Abbott Laboratories, Abbott Park, IL, USA), HCV RNA, and HCV genotype (Abbott RealT*ime* HCV Genotype II, Abbott Laboratories, Abbott Park, IL, USA) were assessed [36]. The upper limits of normal (ULN) AST and ALT levels were 30 U/L for men and 19 U/L for women [37]. We also calculated the AST-to-platelet ratio index (APRI) and fibrosis index based on four parameters (FIB-4) for all patients [38,39]. LSM was performed with the patients lying in a supine position with their right arms tucked behind the head. The probe was placed on the skin of the right intercostal space at the level of the right hepatic lobe. The results of LSM were expressed in kPa with a median value and interquartile range (IQR) of at least 10 valid measurements and a successful rate of more than 60%. LSM failure was defined as a zero valid measurement, and unreliable examinations were defined as less than 10 valid measurements, a successful rate of less than 60%, or the IQR of more than 30% of the median LSM value. Patients with an LSM of ≤6.0 kPa, 6.1–7.0 kPa, 7.1–9.4 kPa, 9.5–12.4 kPa, and ≥12.5 had a fibrosis stage of F0, F1, F2, F3, and F4, respectively [40].

SAPI was measured by placing the ultrasound probe on the skin of the left intercostal space and sampling the signals in the main branches of the intrasplenic arteries at the splenic hilum. The SAPI was calculated using the following formula: (peak systolic velocity—end-diastolic velocity)/mean velocity [35].

### 2.3. Statistical Analysis

All statistical analyses were performed using the Statistical Program for Social Sciences (SPSS Statistics Version 26.0, IBM Corp., Armonk, NY, USA). Baseline characteristics were shown as a median (range) and number (percentage) when appropriate. We analyzed the relationship between SAPI and LSM with the Pearson correlation. Furthermore, we analyzed the relationship between SAPI and different hepatic fibrosis stages (F0, F1, F2, F3, and F4) with Spearman’s rank correlation. The receiver operating characteristic (ROC) curves to predict patients with a fibrosis stage of ≥F1, ≥F2, ≥F3, and F4 were constructed for SAPI, APRI, and FIB-4. The areas under the ROC curves (AUROCs) with a 95% confidence interval (CI) of SAPI, APRI, and FIB-4 were shown according to different fibrosis stages [41]. The Youden index with a maximal value (sensitivity + specificity − 1) was selected to distinguish different fibrosis stages. All statistics were two-tailed, and the results with a *p*-value < 0.05 were considered statistically significant.

## 3. Results

### 3.1. Patient Characteristics

**Figure 1:**
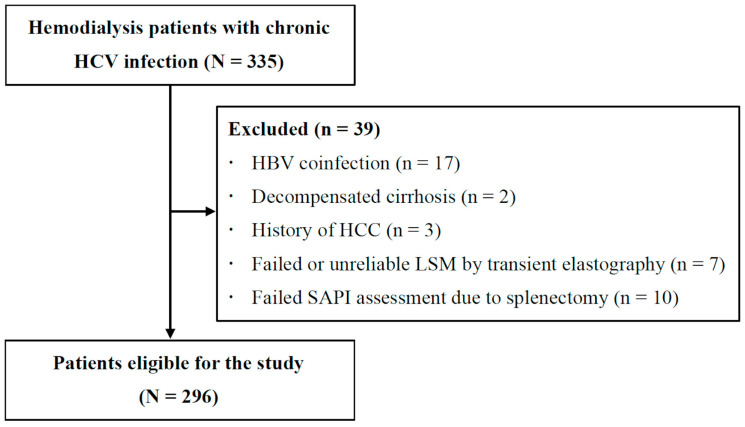
Study flow.

Of 335 hemodialysis patients with chronic HCV infection, 296 were eligible for the study after excluding 39 because of HBV coinfection (*n* = 17), decompensated cirrhosis (*n* = 2), a history of HCC (*n* = 3), a failed or unreliable LSM (*n* = 7), or a failed SAPI assessment due to splenectomy (Figure 1).

The baseline characteristics are shown in Table 1. The median age was 55, and 186 (62.8%) were males. Two hundred and six (69.6%) and seventy-four (25.0%) were infected with HCV genotype 1 and genotype 2. The median LSM was 6.7 kPa, and the median level of SAPI was 1.09. Regarding the stage of hepatic fibrosis, 108 (36.5%), 61 (20.6%), 65 (22.0%), 30 (10.1%), and 32 (10.8%) patients had a fibrosis stage of F0, F1, F2, F3, and F4, respectively. The median levels of APRI and FIB-4 were 0.69 and 1.67.

### 3.2. Correlation between SAPI and LSM and Stage of Hepatic Fibrosis

**Figure 2:**
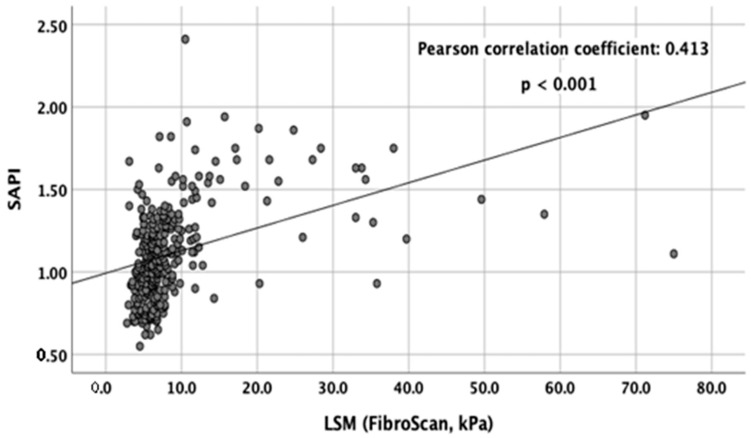
Scatter plot of SAPI and LSM (kPa) with TE. The Pearson correlation coefficient was 0.413 (*p* < 0.001).

**Figure 3:**
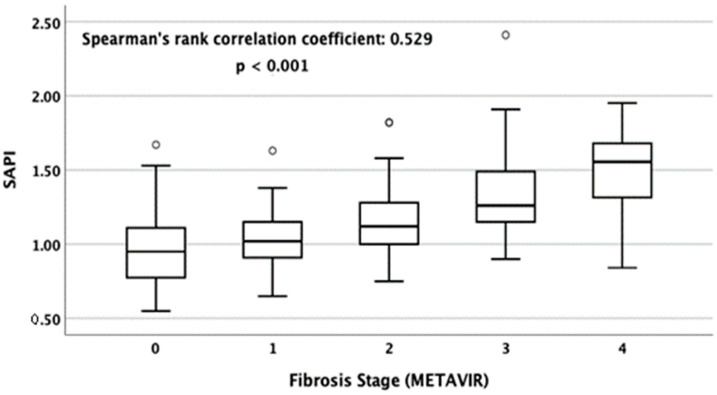
Box plots of SAPI for METAVIR fibrosis stages F0, F1, F2, F3, and F4 as determined using LSM. The tops and bottoms of the boxes are the first and the third quartiles. The tops and bottoms of the horizontal lines are the upper and lower whiskers. The circles denote mild outliers. The Spearman’s rank correlation coefficient was 0.529 (*p* < 0.001).

The levels of SAPI were significantly correlated with LSMs (Pearson correlation coefficient: 0.413, *p* < 0.001) (Figure 2). Figure 3 shows the box plots of SAPI according to different METAVIR fibrosis stages assessed using TE. The median (IQR) levels of SAPI for F0, F1, F2, F3, and F4 were 0.95 (0.77–1.12), 1.02 (0.91–1.16), 1.12 (1.00–1.29), 1.26 (1.15–1.50), and 1.56 (1.31–1.68), respectively. The Spearman’s rank correlation coefficient between SAPI and the stage of hepatic fibrosis was 0.529 (*p* < 0.001).

### 3.3. AUROCs of SAPI to Predict the Severity of Hepatic Fibrosis

**Figure 4:**
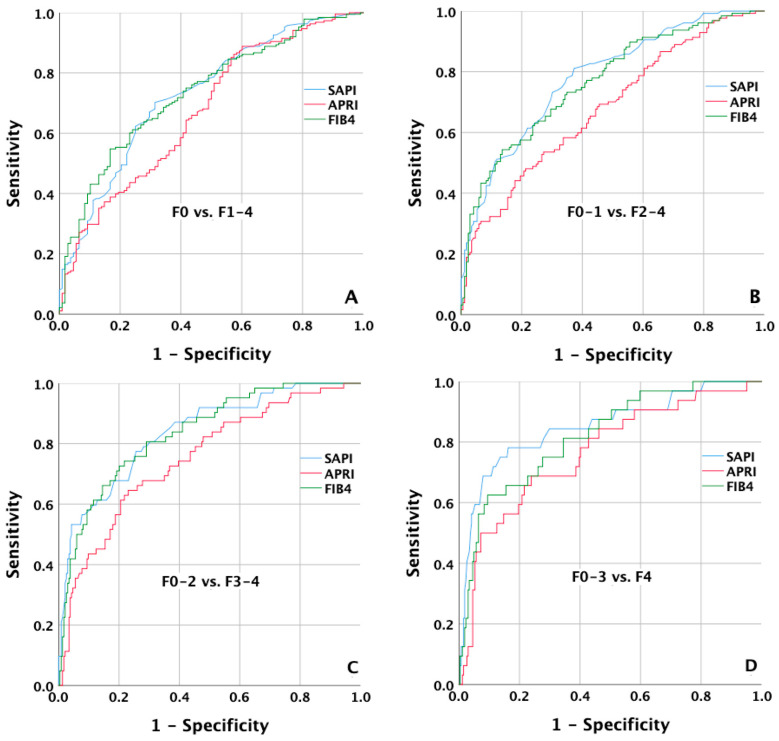
ROC curves of SAPI to predict patients with a fibrosis stage of ≥F1, ≥F2, ≥F3, and F4. The AUROCs of SAPI were (**A**) 0.730 (95% CI: 0.671–0.789) for ≥F1; (**B**) 0.782 (95% CI: 0.730–0.834) for ≥F2; (**C**) 0.838 (95% CI: 0.781–0.894) for ≥F3; (**D**) 0.851 (95% CI: 0.771–0.931) for F4, respectively.

The ROC curves of SAPI, APRI, and FIB-4 according to different stages of hepatic fibrosis are shown in Figure 4. To predict patients with a fibrosis stage of ≥F1, the AUROCs of SAPI, APRI, and FIB-4 were 0.730 (95% CI: 0.671–0.789), 0.674 (95% CI: 0.611–0.738), and 0.735 (95% CI: 0.678–0.793). To predict patients with a fibrosis stage of ≥F2, the AUROCs of SAPI, APRI, and FIB-4 were 0.782 (95% CI: 0.730–0.834), 0.680 (95% CI: 0.619–0.741), and 0.768 (95% CI: 0.714–0.822). To predict patients with a fibrosis stage of ≥F3, the AUROCs of SAPI, APRI, and FIB-4 were 0.838 (95% CI: 0.781–0.894), 0.751 (95% CI: 0.682–0.820), and 0.836 (95% CI: 0.781–0.890). To predict patients with a fibrosis stage of F4, the AUROCs of SAPI, APRI, and FIB-4 were 0.851 (95% CI: 0.771–0.931), 0.766 (95% CI: 0.674–0.857), and 0.822 (95% CI: 0.745–0.898) (Table 2).

### 3.4. Selective Cutoff Values for SAPI to Predict the Severity of Hepatic Fibrosis

The maximal Youden indices of SAPI to predict patients with a fibrosis stage of ≥F1, ≥F2, ≥F3, and F4 were 1.04, 1.06, 1.19, and 1.30, respectively. The sensitivity was 70.2%, 78.2%, 77.4%, and 78.1% to predict ≥F1, ≥F2, ≥F3, and F4, respectively. The specificity to predict a fibrosis stage of ≥F1, ≥F2, ≥F3, and F4 was 68.5%, 65.1%, 74.4%, and 83.7%, respectively. The PPVs were 79.5%, 62.7%, 44.4%, and 36.7%, and the NPVs were 56.9%, 79.8%, 92.6%, and 96.9% to predict patients with a fibrosis stage of ≥F1, ≥F2, ≥F3, and F4, respectively. The diagnostic accuracies for a fibrosis stage of ≥F1, ≥F2, ≥F3, and F4 were 69.6%, 67.2%, 75.0% and 83%, respectively (Table 3).

## 4. Discussion

The noninvasive tools to stage hepatic fibrosis in hemodialysis patients with HCV infection are expected to prevail in clinical practice because liver biopsy is invasive and associated with various complications [27]. Our study demonstrated that in hemodialysis patients with chronic HCV infection, the levels of SAPI tended to increase with increasing LSMs and stage of hepatic fibrosis. By comparing the two commonly used biochemical indices, the APRI and FIB-4, we revealed that the overall diagnostic power of SAPI was similar to FIB-4 but was superior to APRI in staging hepatic fibrosis in our patients. In nonuremic patients with HCV, FIB-4 has been shown to perform better than APRI in distinguishing the severity of hepatic fibrosis [29,40]. We also observed a superior diagnostic power of FIB-4 over APRI to stage hepatic fibrosis in hemodialysis patients with chronic HCV infection.

In our study, the AUROCs of SAPI increased with more severe hepatic fibrosis. When we examined the maximal Youden index to diagnose a fibrosis stage of ≥F1, ≥F2, ≥F3, and F4, we found that the main power of SAPI was to diagnose patients with ≥F1 to reach a PPV of 79.5% at a cutoff value of 1.04, and patients with ≥F2, ≥F3, and F4 with NPVs of 79.8%, 92.6%, and 96.9%, respectively, at cutoff values of 1.06, 1.19, and 1.30. Using these cutoff values, physicians can correctly diagnose the stage of hepatic fibrosis in more than two-thirds of hemodialysis patients with chronic HCV infection [42]. Although SAPI was considered a valuable index to predict the severity of hepatic fibrosis in hemodialysis patients with chronic HCV infection, the diagnostic performance of SAPI seemed to be inferior to nonuremic patients with HCV, which revealed ARUOCs of 0.86 to 0.89 to predict a fibrosis stage of ≥F2, and 0.90 to 0.92 to predict a fibrosis stage of F4 [29,30,31]. Because body fluid status may significantly affect portal hemodynamics in hemodialysis patients, we speculated that the efficiency of hemodialysis might contribute to the lower diagnostic accuracies of SAPI in predicting the stage of hepatic fibrosis [43,44].

Because TE may misclassify the stage of hepatic fibrosis in a small proportion of hemodialysis patients with chronic HCV infection, prior studies have demonstrated that the diagnostic accuracies of APRI and FIB-4 to stage hepatic fibrosis were better if they took percutaneous liver biopsy rather than LSM to be the reference standard [40,45,46,47]. Based on the similar AUROCs of APRI and FIB-4 between our and prior reports using TE as the reference standard, we confirmed that SAPI had comparable diagnostic accuracies to FIB-4 and was superior to APRI to assess the stage of hepatic fibrosis in hemodialysis patients with chronic HCV infection [47,48,49].

Compared to computed tomography (CT) or magnetic resonance imaging (MRI)-based techniques to assess the hepatic fibrosis, most physicians can easily complete the measurement of SAPI within 5–10 min using ultrasonographic machines equipped with the automatic Doppler tracing function through placing the cursor on the main branches of the splenic artery [31]. Furthermore, DDU can be concomitantly performed at routine gray-scale ultrasonographic HCC surveillance without additional costs and concerns for radiation or contrast-related injuries with CT or MRI. While SAPI may play a role in improving the diagnostic yield of hepatic fibrosis in hemodialysis patients with chronic HCV infection, further studies should target cost-effectiveness and the cost–utility of combining SAPI, FIB-4, and LSM to optimize the care of these patients.

To our knowledge, this study was the first to assess the clinical utility of SAPI, an easily-performed DDU analysis, to diagnose the stage of hepatic fibrosis in hemodialysis patients with chronic HCV infection. The strengths of our study include (1) a sizable number of hemodialysis patients in this analysis; and (2) a homogeneous population excluding HBV or HIV infection, decompensated cirrhosis, or a history of HCC. However, our study has some limitations. First, this retrospective study could not standardize the patients’ fluid status during the SAPI assessment. Second, we were unable to assess the intra- and inter-observer variations of SAPI assessment due to the retrospective nature of this study. However, all SAPI measurements in our study were performed by well-trained physicians, who demonstrated low intra- and inter-observer variations in previous reports [26,28]. Third, we did not adopt a percutaneous liver biopsy, an invasive procedure seldom performed for hemodialysis patients due to the concerns of bleeding events, as the reference standard.

In conclusion, our study demonstrates that SAPI is a useful noninvasive index to stage the severity of hepatic fibrosis in hemodialysis patients with chronic HCV infection. The diagnostic performance of SAPI is comparable to FIB-4 and superior to APRI. Using the maximal Youden indices for SAPI, the stage of hepatic fibrosis can be correctly diagnosed in more than two-thirds of hemodialysis patients with chronic HCV infection. Independent studies are needed to validate the value of SAPI to predict the stage of hepatic fibrosis in this special population.

## Figures and Tables

**Table 1:** Baseline characteristics.

Characteristics ^a^	Patient (N = 296)
Age, year	55 (23–81)
Male, *n* (%)	186 (62.8)
HCV RNA, log_10_, IU/mL	5.80 (1.83–8.00)
HCV genotype, (%)	
1a	17 (5.7)
1b	189 (63.9)
2	74 (25.0)
6	7 (2.4)
Mixed	8 (2.7)
Indeterminate	1 (0.3)
LSM, kPa ^b^	6.7 (2.8–75.0)
Fibrosis stage (METAVIR), *n* (%) ^c^	
F0	108 (36.5)
F1	61 (20.6)
F2	65 (22.0)
F3	30 (10.1)
F4	32 (10.8)
SAPI	1.09 (0.55–2.41)
BMI, kg/m^2^	22.7 (14.9–37.2)
Hemoglobin, g/dL	11.7 (7.9–17.2)
White blood cell count, 10^9^ cells/L	5.7 (1.2–11.6)
Platelet count, 10^9^/L	180 (39–432)
Albumin, g/dL	4.3 (2.2–5.4)
Total bilirubin, mg/dL	0.5 (0.1–1.6)
AST, ULN ^d^	1.2 (0.3–13.7)
ALT, ULN ^d^	1.4 (0.1–24.7)
APRI	0.69 (0.14–8.45)
FIB-4 ^e^	1.67 (0.32–10.74)
eGFR, mg/dL/1.73 m^2 e^	6 (2–14)

HCV, hepatitis C virus; RNA, ribonucleic acid; LSM, liver stiffness measurement; kPa, kilo Pascal; SAPI, splenic arterial pulsatility index; BMI, body mass index; AST, aspartate transaminase; ALT, alanine transaminase; ULN, upper limit of normal; APRI, AST-to-platelet ratio index; FIB-4, fibrosis index based on four parameters; eGFR, estimated glomerular filtration rate. ^a^ Data are shown as a median (range) unless otherwise indicated. ^b^ Assessed using TE. ^c^ The LSM cutoff values for a hepatic fibrosis stage of F0, F1, F2, F3, and F4 are ≤6.0 kPa, 6.1–7.0 kPa, 7.1–9.4 kPa, 9.5–12.4 kPa, and ≥12.5 kPa, respectively. ^d^ The upper limit of normal (ULN) is 30 U/L for men and 19 U/L for women. ^e^ Assessed using the chronic kidney disease epidemiology collaboration (CKD-EPI) equation.

**Table 2:** The areas under the receiver operating characteristics (AUROCs) of SAPI, APRI, and FIB-4 to predict different stages of hepatic fibrosis.

Stage of Hepatic Fibrosis	SAPI		APRI		FIB-4	
	AUROC	95% CI	AUROC	95% CI	AUROC	95% CI
≥F1	0.730	0.671–0.789	0.674	0.611–0.738	0.735	0.678–0.793
≥F2	0.782	0.730–0.834	0.680	0.619–0.741	0.768	0.714–0.822
≥F3	0.838	0.781–0.894	0.751	0.682–0.820	0.836	0.781–0.890
F4	0.851	0.771–0.931	0.766	0.674–0.857	0.822	0.745–0.898

SAPI, splenic arterial pulsatility index; APRI, AST-to-platelet ratio index; FIB-4, fibrosis index based on four parameters; AUROC, area under the receiver operating characteristics; CI, confidence interval.

**Table 3:** The maximal Youden indices of SAPI to predict different stages of hepatic fibrosis.

SAPI ^a^	Fibrosis Stage	All Patients (N = 296)			Sensitivity (%)	Specificity (%)	PPV (%)	NPV (%)	Accuracy (%)
		Tested Positive, *n* (%)	Actual Positive, *n* (%)	Actual Negative, *n* (%)					
1.04	≥F1	166 (56.1)	188 (63.5)	108 (36.5)	70.2	68.5	79.5	56.9	69.6
1.06	≥F2	148 (50.0)	127 (42.9)	169 (57.1)	78.0	65.1	62.7	79.8	67.2
1.19	≥F3	108 (36.5)	62 (20.9)	234 (79.1)	77.4	74.4	44.4	92.6	75.0
1.30	F4	68 (23.0)	32 (10.8)	264 (89.2)	78.1	83.7	36.7	96.9	83.1

SAPI, splenic arterial pulsatility index; PPV, positive predictive value; NPV, negative predictive value. ^a^ Youden index is defined as sensitivity + specificity − 1.

## Data Availability

Data for this study, though not available in a public repository, can be made available upon reasonable request.

## References

[1] Jadoul M., Bieber B.A., Martin P., Akiba T., Nwankwo C., Arduino J.M., Goodkin D.A., Pisoni R.L. (2019). Prevalence, incidence, and risk factors for hepatitis C virus infection in hemodialysis patients. Kidney Int..

[2] Polaris Observatory HCV Collaborators (2022). Global change in hepatitis C virus prevalence and cascade of care between 2015 and 2020: A modelling study. Lancet Gastroenterol. Hepatol..

[3] Liu C.-H., Kao J.-H. (2022). Pan-genotypic direct-acting antivirals for patients with hepatitis C virus infection and chronic kidney disease stage 4 or 5. Hepatol. Int..

[4] Okuda K., Hayashi H., Kobayashi S., Irie Y. (1995). Mode of hepatitis C infection not associated with blood transfusion among chronic hemodialysis patients. J. Hepatol..

[5] Okuda K., Kanda T., Yokosuka O., Hayashi H., Yokozeki K., Ohtake Y., Irie Y. (1997). GB virus-C infection among chronic haemodialysis patients: Clinical implications. J. Gastroenterol. Hepatol..

[6] Okuda K., Yokosuka O. (2004). Natural history of chronic hepatitis C in patients on hemodialysis: Case control study with 4–23 years of follow-up. World J. Gastroenterol..

[7] Fabrizi F., Takkouche B., Lunghi G., Dixit V., Messa P., Martin P. (2007). The impact of hepatitis C virus infection on survival in dialysis patients: Meta-analysis of observational studies. J. Viral Hepat..

[8] Fabrizi F., Dixit V., Messa P. (2012). Impact of hepatitis C on survival in dialysis patients: A link with cardiovascular mortality?. J. Viral Hepat..

[9] Goodkin D.A., Bieber B., Jadoul M., Martin P., Kanda E., Pisoni R.L. (2017). Mortality, hospitalization, and quality of life among patients with hepatitis C infection on hemodialysis. Clin. J. Am. Soc. Nephrol..

[10] Söderholm J., Millbourn C., Büsch K., Kövamees J., Schvarcz R., Lindahl K., Bruchfeld A. (2018). Higher risk of renal disease in chronic hepatitis C patients: Antiviral therapy survival benefit in patients on hemodialysis. J. Hepatol..

[11] Goodkin D.A., Bieber B., Gillespie B., Robinson B.M., Jadoul M. (2013). Hepatitis C infection is very rarely treated among hemodialysis patients. Am. J. Nephrol..

[12] Liu C.-H., Huang C.-F., Liu C.-J., Dai C.-Y., Liang C.-C., Huang J.-F., Hung P.-H., Tsai H.-B., Tsai M.-K., Chen S.-I. (2013). Pegylated interferon-α2a with or without low-dose ribavirin for treatment-naive patients with hepatitis C virus genotype 1 receiving hemodialysis: A randomized trial. Ann. Intern. Med..

[13] Liu C.-H., Liu C.-J., Huang C.-F., Lin J.-W., Dai C.-Y., Liang C.-C., Huang J.-F., Hung P.-H., Tsai H.-B., Tsai M.-K. (2015). Peginterferon alfa-2a with or without low-dose ribavirin for treatment-naive patients with hepatitis C virus genotype 2 receiving haemodialysis: A randomised trial. Gut.

[14] Pockros P.J., Reddy K.R., Mantry P.S., Cohen E., Bennett M., Sulkowski M.S., Bernstein D.E., Cohen D.E., Shulman N.S., Wang D. (2016). Efficacy of direct-acting antiviral combination for patients with hepatitis C virus genotype 1 infection and severe renal impairment or end-stage renal disease. Gastroenterology.

[15] Roth D., Nelson D.R., Bruchfeld A., Liapakis A., Silva M., Monsour H., Martin P., Pol S., Londoño M.-C., Hassanein T. (2015). Grazoprevir plus elbasvir in treatment-naive and treatment-experienced patients with hepatitis C virus genotype 1 infection and stage 4–5 chronic kidney disease (the C-SURFER study): A combination phase 3 study. Lancet.

[16] Gane E., Lawitz E., Pugatch D., Papatheodoridis G., Bräu N., Brown A., Pol S., Leroy V., Persico M., Moreno C. (2017). Glecaprevir and pibrentasvir in patients with HCV and severe renal impairment. N. Engl. J. Med..

[17] Lawitz E., Flisiak R., Abunimeh M., Sise M.E., Park J.Y., Kaskas M., Bruchfeld A., Wörns M.-A., Aglitti A., Zamor P.J. (2020). Efficacy and safety of glecaprevir/pibrentasvir in renally impaired patients with chronic HCV infection. Liver Int..

[18] Liu C.-H., Yang S.-S., Peng C.-Y., Lin W.-T., Liu C.-J., Su T.-H., Tseng T.-C., Chen P.-J., Chen D.-S., Kao J.-H. (2020). Glecaprevir/pibrentasvir for patients with chronic hepatitis C virus infection and severe renal impairment. J. Viral Hepat..

[19] Borgia S.M., Dearden J., Yoshida E.M., Shafran S.D., Brown A., Ben-Ari Z., Cramp M.E., Cooper C., Foxton M., Rodriguez C.F. (2019). Sofosbuvir/velpatasvir for 12 weeks in hepatitis C virus-infected patients with end-stage renal disease undergoing dialysis. J. Hepatol..

[20] Liu C.-H., Chen C.-Y., Su W.-W., Tseng K.-C., Lo C.-C., Liu C.-J., Chen J.-J., Peng C.-Y., Shih Y.-L., Yang S.-S. (2022). Sofosbuvir/velpatasvir with or without low-dose ribavirin for patients with chronic hepatitis C virus infection and severe renal impairment. Gut.

[21] Kim N.J., Vutien P., Cleveland E., Cravero A., Ioannou G.N. (2022). Fibrosis stage-specific incidence of hepatocellular cancer after hepatitis C cure with direct-acting antivirals: A systematic review and meta-analysis. Clin. Gastroenterol. Hepatol..

[22] Bravo A.A., Sheth S.G., Chopra S. (2001). Liver biopsy. N. Engl. J. Med..

[23] Pawa S., Ehrinpreis M., Mutchnick M., Janisse J., Dhar R., Siddiqui F.A. (2007). Percutaneous liver biopsy is safe in chronic hepatitis C patients with end-stage renal disease. Clin. Gastroenterol. Hepatol..

[24] Cotler S.J., Diaz G., Gundlapalli S., Jakate S., Chawla A., Mital D., Jensik S., Jensen D.M. (2002). Characteristics of hepatitis C in renal transplant candidates. J. Clin. Gastroenterol..

[25] McGill D.B., Rakela J., Zinsmeister A.R., Ott B.J. (1990). A 21-year experience with major hemorrhage after percutaneous liver biopsy. Gastroenterology.

[26] Maharaj B., Leary W.P., Naran A.D., Maharaj R.J., Cooppan R.M., Pirie D., Pudifin D.J. (1986). Sampling variability and its influence on the diagnostic yield of percutaneous needle biopsy of the liver. Lancet.

[27] Liu C.-H., Kao J.-H. (2022). Noninvasive diagnosis of hepatic fibrosis in hemodialysis patients with hepatitis C virus infection. Diagnostics.

[28] Bolognesi M., Sacerdoti D., Merkel C., Gerunda G., Maffei-Faccioli A., Angeli P., Jemmolo R.M., Bombonato G., Gatta A. (1996). Splenic Doppler impedance indices: Influence of different portal hemodynamic conditions. Hepatology.

[29] Liu C.-H., Lin J.-W., Tsai F.-C., Yang P.-M., Lai M.-Y., Chen J.-H., Kao J.-H., Chen D.-S. (2006). Noninvasive tests for the prediction of significant hepatic fibrosis in hepatitis C virus carriers with persistently normal alanine aminotransferases. Liver Int..

[30] Ullah H., Rehman A.-U., Aslam H. (2018). Diagnostic accuracy of splenic arterial pulsatility index in predicting fibrosis associated with chronic hepatitis C. Pak. Armed Forces Med. J..

[31] Liu C.-H., Hsu S.-J., Lin J.-W., Hwang J.-J., Liu C.-J., Yang P.-M., Lai M.-Y., Chen P.-J., Chen J.-H., Kao J.-H. (2007). Noninvasive diagnosis of hepatic fibrosis in patients with chronic hepatitis C by splenic Doppler impedance index. Clin. Gastroenterol. Hepatol..

[32] Nishimura T., Iijima H., Nishikawa H., Kondo R., Yano H., Kage M., Aoki T., Nakano C., Yuri Y., Ishii N. (2019). Liver fibrosis markers as assessed by ultrasound elastography and serum samples: A large comparative study in hepatitis virus B and C liver diseases. Hepatol. Res..

[33] Levey A.S., Stevens L.A., Schmid C.H., Zhang Y.L., Castro A.F., Feldman H.I., Kusek J.W., Eggers P., Van Lente F., Greene T. (2009). A new equation to estimate glomerular filtration rate. Ann. Intern. Med..

[34] Liu C.-H., Lee M.-H., Lin J.-W., Liu C.-J., Su T.-H., Tseng T.-C., Chen P.-J., Chen D.-S., Kao J.-H. (2020). Evolution of eGFR in chronic HCV patients receiving sofosbuvir-based or sofosbuvir-free direct-acting antivirals. J. Hepatol..

[35] Liu C.-H., Lin J.-W., Liu C.-J., Su T.-H., Wu J.-H., Tseng T.-C., Chen P.-J., Kao J.-H. (2023). Long-term evolution of estimated glomerular filtration rate in patients with antiviral treatment for hepatitis C virus infection. Clin. Gastroenterol. Hepatol..

[36] Liu C.-H., Liang C.-C., Liu C.-J., Lin C.-L., Su T.-H., Yang H.-C., Chen P.-J., Chen D.-S., Kao J.-H. (2015). Comparison of Abbott RealTime HCV Genotype II with Versant line probe assay 2.0 for hepatitis C virus genotyping. J. Clin. Microbiol..

[37] Prati D., Taioli E., Zanella A., Della Torre E., Butelli S., Del Vecchio E., Vianello L., Zanuso F., Mozzi F., Milani S. (2002). Updated definitions of healthy ranges for serum alanine aminotransferase levels. Ann. Intern. Med..

[38] Wai C.-T., Greenson J.K., Fontana R.J., Kalbfleisch J.D., Marrero J.A., Conjeevaram H.S., Lok A.S.-F. (2003). A simple noninvasive index can predict both significant fibrosis and cirrhosis in patients with chronic hepatitis C. Hepatology.

[39] Sterling R.K., Lissen E., Clumeck N., Sola R., Correa M.C., Montaner J., Sulkowski M.S., Torriani F.J., Dieterich D.T., Thomas D.L. (2006). Development of a simple noninvasive index to predict significant fibrosis in patients with HIV/HCV coinfection. Hepatology.

[40] Liu C.-H., Liang C.-C., Huang K.-W., Liu C.-J., Chen S.-I., Lin J.-W., Hung P.-H., Tsai H.-B., Lai M.-Y., Chen P.-J. (2011). Transient elastography to assess hepatic fibrosis in hemodialysis chronic hepatitis C patients. Clin. J. Am. Soc. Nephrol..

[41] Hanley J.A., McNeil B.J. (1983). A method of comparing the areas under receiver operating characteristic curves derived from the same cases. Radiology.

[42] Trevizoli J.E., de Paula Menezes R., Ribeiro Velasco L.F., Amorim R., de Carvalho M.B., Mendes L.S., Neto C.J., de Deus Macedo J.R., de Assis F., Neves R. (2008). Hepatitis C is less aggressive in hemodialysis patients than in nonuremic patients. Clin. J. Am. Soc. Nephrol..

[43] Khurana S., Simcox T., Twaddell W., Drachenberg C., Flasar M. (2010). Dialysis reduces portal pressure in patients with chronic hepatitis C. Artif. Organs.

[44] Ergun T., Lakadamyali H. (2010). Doppler ultrasound evaluation of morphological and hemodynamical changes of hepatic and mesenteric structures in end-stage renal disease patients on regular hemodialysis. Int. Urol. Nephrol..

[45] Schiavon L.L., Schiavon J.L.N., Filho R.J.C., Sampaio J.P., Lanzoni V.P., Silva A.E.B., Ferraz M.L.G. (2007). Simple blood tests as noninvasive markers of liver fibrosis in hemodialysis patients with chronic hepatitis C virus infection. Hepatology.

[46] Liu C.-H., Liang C.-C., Liu C.-J., Hsu S.-J., Lin J.-W., Chen S.-I., Hung P.-H., Tsai H.-B., Lai M.-Y., Chen P.-J. (2010). The ratio of aminotransferase to platelets is a useful index for predicting hepatic fibrosis in hemodialysis patients with chronic hepatitis C. Kidney Int..

[47] Pestana N.F., Equi C.M.A., Gomes C.P., Cardoso A.C., Zumack J.P., Villela-Nogueira C.A., Perez R.M. (2021). Aminotransferase-to-platelet ratio index and Fibrosis-4 index score predict hepatic fibrosis evaluated by transient hepatic elastography in hepatitis C virus-infected hemodialysis patients. Eur. J. Gastroenterol. Hepatol..

[48] Lee J.-J., Wei Y.-J., Lin M.-Y., Niu S.-W., Hsu P.-Y., Huang J.-C., Jang T.-Y., Yeh M.-L., Huang C.-I., Liang P.-C. (2020). The applicability of non-invasive methods for assessing liver fibrosis in hemodialysis patients with chronic hepatitis C. PLoS ONE.

[49] Nakano C., Nishimura T., Tada T., Yoshida M., Takashima T., Aizawa N., Ikeda N., Nishikawa H., Enomoto H., Hatano E. (2021). Severity of liver fibrosis using shear wave elastography is influenced by hepatic necroinflammation in chronic hepatitis patients, but not in cirrhotic patients. Hepatol. Res..

